# Treatment of Cold Abscess by Iodoform

**Published:** 1890-12-27

**Authors:** 


### TREATMENT OF COLD ABSCESS BY IODOFORM.

We have so frequently referred to this subject that we should not have mentioned another case, were it not that the success that followed the injections was the more remarkable from the fact that the abscess, which had existed for many months, and at length involved nearly the whole of the right side of the chest, had its origin in caries and tubercular disease of the upper part of the sternum and the articular ends of the three upper ribs. The patient resolutely refusing to submit to excision of the diseased bone, Dr. Kerschner opened the abscess, evacuating a pint and a half of pus, and injected 1½ oz. of a solution of iodoform 10 parts, glycerine 50, and water 100. The injection was repeated twice, each time under strict antiseptic precautions, and in a fortnight the abscess was completely healed, the iodoform having doubtless destroyed the vitality of the tubercular bacilli, and thus arrested the disease in the bones. Dr. Kerschner finds glycerine a far more efficient solvent of iodoform than olive oil, which Klingemann has shown not to dissolve more than 3 per cent.; the greater part of the iodoform in the 10 or 20 per cent. solutions (?) commonly recommended being merely suspended in the oil.

